# Observations on the Powers and Effects of Cold as a Cause of Disease; with Some Remarks on the Best Means of Preventing Its Morbid Effects, &c.

**Published:** 1832-06

**Authors:** John Clendinning

**Affiliations:** Edinburgh and Oxford, Fellow and Censor of the Royal College of Physicians of London, and Physician to the Western Dispensary.


THE LONDON
### Medical and Physical Journal.

400, VOL. LXVII.] JUNE 1832. [72, *New Series.*

"For many fortunate discoveries in medicine, and for the detection of numerous errors, the world is indebted to the rapid circulation of Monthly Journals; and there never existed any work to which the Faculty, in Europe and America, were under deeper obligations than to the 'Medical and Physical Journal of London,' now forming a long but invaluable series."—RUSH.

### ORIGINAL PAPERS, AND CASES

### OBTAINED FROM PUBLIC INSTITUTIONS AND OTHER AUTHENTIC SOURCES.

### COLD AS A CAUSE OF DISEASE.

*Observations on the Powers and Effects of Cold as a Cause of Disease; with some Remarks on the best Means of Preventing its Morbid Effects, &c.* By JOHN CLENDINNING, M.D. of Edinburgh and Oxford, Fellow and Censor of the Royal College of Physicians of London, and Physician to the Western Dispensary.

THERE is no one of the causes of disease which, in popular estimation, is so extensively mischievous as cold. Febrile, inflammatory, almost universally, and in numberless instances nervous, cachectic, and other forms of disease, are laid to the charge of that agent; and the popular opinion has unquestionably a foundation in truth, and is to a great extent confirmed by daily professional experience. A very large portion of the mortality of all climates is attributable to changes of atmospherical heat and humidity, and this in a double sense; much disease is occasioned by hourly and daily fluctuations of the thermometer and hygrometer at all seasons; while a still greater destruction of human life is found to depend on the change from the soft airs of summer to the chilling blasts of winter and spring. "Half of mankind," says RITTER, "perish before the close of the third year."—"If, on the one hand," says the same writer, in the article *Ausdünstung* of Ersch and Gruber's Encyclopædia, "we recollect the susceptibility of the teguments and the frailty of the incompletely formed organs, and, on the other hand, the numberless occasions of taking cold, from baptism forward to the time when the child can wallow in the puddle, and sit down to cool itself in the blast, we shall soon cease to wonder at the amount of infant mortality from cold."—"The contagious class excepted," he says in another place, "there is no disease, "not even of the nails, hair, or bones, that cold may not occasion."—"With the exception of a small number of diseases occasioned by unwholesome occupations and by the contagious, the great mass of human malady in this metropolis," says the observant BATEMAN, "is referrible to the climate or state of the seasons, and to intemperance; but that, of these two causes, the vicissitudes of the weather, especially its cold, are by far the most prolific sources of mischief." (*Diseases of London.*)

But it is the change from the mild to the severe season that, in modern times, proves the most deleterious to life and health. We have learnt how to check the production and limit the range of those destructive emanations, animal and terrestrial, that caused such devastations amongst our predecessors of earlier ages: but climate, and the causes of atmospherical changes, continue beyond our control; winter is the great enemy of the very young and the very old. "The increased mortality occasioned by severe winters has generally fallen heaviest," says Bateman, "on the aged and young children, whose vital powers are possessed of less energy than during the intermediate periods of life." Nor are the healthy without their share of danger and suffering. "Those who erroneously think the cold of winter bracing and wholesome, (and the error is unfortunately very general,) forget what the weak and infirm, the aged and invalid, suffer under such circumstances, and do not reflect," says Bateman, "on the multiplying brood of pulmonary, rheumatic, dysenteric, and other inflammatory disorders, with consumption at their head, from which even the most robust are not exempt."—"During the winter of 1814," he says again, "in which the frost continued three months, the number of patients at the public dispensaries exceeded by seven hundred the ordinary average; in other years, for the same period, not much exceeding five hundred. The bills of mortality exhibited a corresponding increase at that time; and an attentive observer could not fail to remark, during the whole spring of that year, how much the gaiety of that season was chequered by the numerous funerals which daily passed along the streets." The January of 1795 was, it is well known, the coldest, while that of the following year (viz. 1796) was the warmest, January of which any regular account has been kept in this country. From the bills of mortality of those years, Dr. HEBERDEN ascertained that the whole mortality of January 1795 was double that of the January following; and that the mortality amongst the aged in particular was five times larger in the former month than in the latter. Dr. Heberden discovered likewise a great relative increase of mortality amongst infants and young children. Like results have been lately obtained by Dr. Milne Edwards, in Paris, in researches of which I have not at hand the particulars; and these facts and opinions entirely coincide with what I have myself adopted from observation, on a considerable scale, amongst the poor of Westminster.

Winter and spring invariably make great additions to the sufferings of invalids, particularly in pectoral complaints, as well as great inroads on the general previously healthy community. I have had frequent opportunity of observing an increase and aggravation of rheumatic and pulmonic diseases in particular, in consequence of a change of wind from the south and south-west towards the northern and north-eastern quarters.

The remaining matter of this portion of the medicinal history of cold I shall distribute into six sections:

1. Definition of terms.
2. Morbific properties of cold.
3. Diseases of cold.
4. Principal forms of morbific cold.
5. Circumstances most favorable to the morbific action of cold.
6. The best means of preventing diseases of cold.

*Definition of Cold, &c.*

In medicine, cold means either a familiar disease or a well-known disagreeable sensation, or else a temperature of surrounding bodies considerably less than that of the more perfect animals, and capable of causing in them the peculiar sensation above referred to, and ultimately, if applied under favorable circumstances, of disturbing health, and even of destroying life. We can scarcely fix on any thermometrical degree as the boundary between cold in the last sense, and warmth or heat. Numerous causes modify, in various ways, our susceptibility of the sensation of cold, and likewise of the morbid effects of exposure to low temperatures. Habit modifies susceptibility. A hot bath at ninety-five degrees would be as hot as many could conveniently bear, yet I know a young gentleman, enjoying vigorous health, a native of Britain, who, from frequent use of the warm bath, can now enjoy himself in water as high as 108 or 110 degrees; a temperature a dozen degrees at least higher than he could some time since have borne: on the other hand, the hardiness of some individuals, as couriers, sailors, &c., and their capability of bearing exposure to cold, is familiar to every one. Constitution must also be taken into account: a bath at seventy-five degrees of Fahrenheit, which to most people would be rather disagreeably cool, was oppressively hot to a lady of Rostan's acquaintance. Health also affects the question: in one class of diseases, we have examples at once of opposite extremes of sensibility to change of temperature and to cold. I mean the neuroses. Of defect of sensibility to a frigid atmosphere, CURRIE gives a striking example in the case of "a young woman, once of the greatest delicacy of frame, who, being struck with madness, lay all night (on one occasion) on a cold floor, with hardly the covering that decency required, when the water was frozen on the table by her, and the milk she was to feed on was a mass of ice." The sensibility of many neuralgic patients to cold and change of temperature, is matter of frequent experience. The nature of the cool substance is likewise important, as well as the mode of its application. Every one has observed the difference between the coldness of air and water of like temperatures, and has also remarked the difference between exposure to cold in a still and in an agitated or progressive medium. The countryman is hardier than the citizen, the highlander than the lowlander, the male than the female, the young than the old, the adult of middle age than infants or grey-headed persons. Hunger, venery, drunkenness, in short, a great number of circumstances, modify the sensibility to temperature and susceptibility of morbific impressions; so that a precise determination of boundary line between cold and warmth is impracticable, and the limits that separate moderate from immoderate cold are equally fluctuating, and from the same variability in the susceptibility of individuals, and a similar liability of any determination of the point that we might attempt, to exceptions and restrictions, at once numerous, minute, and equivocal. Suffice it then to say generally, taking as our standard a vigorous adult of these islands, that a still air not more than sixty or seventy degrees below bloodheat, (viz. ninety-eight to ninety-nine degrees, is moderately cold;) while a tranquil atmosphere falling short of ninety-eight to ninety-nine degrees, by seventy or eighty degrees, or more, is immoderately or extremely cold; and that water, from its superior refrigerating power, would, under similar circumstances, be moderately or immoderately cold at temperatures ten to twenty degrees higher than air.

*Morbific Properties of Cold.*

There can be no doubt that the operation of cold as a morbific cause, is always in the first instance chemical. Refrigeration, or abstraction of caloric, is the only effect necessarily and invariably attendant on the application of a cooling substance. The temperature of the external body may be relatively moderate or excessive, the cooling medium may be applied in a tranquil or in a progressive state; and upon such difference in the refrigerant process, no doubt, may depend very important differences in the morbid effects of cold: still, however, the result of the application of cold depends, to an equal or even greater extent, on the susceptibility of the subject; and that susceptibility is dependent on and constituted by multifarious conditions, with which atmospherical heat or other changes have nothing to do. Refrigeration is then always the first step in the process that ends in a disease of cold; the rest depends on the properties and susceptibilities of each of the principal organs and functions exposed to refrigeration, and to the recuperative energy and stability of the whole frame.

The parts and functions whose physiological states are sensibly modified and vitiated by cold are, 1st, the teguments or skin, and mucous membranes; 2d, the muscles; 3d, the blood; 4th, the respiration: 5th, the caloric function; 6th, the circulation; 7th, the absorption; 8th, the nervous system. I proceed to the enumeration and illustration of the morbid effects of cold on each of those parts and functions: the morbific properties, or modes of noxious action, of that great enemy of life once pointed out, the reader will, I hope, require little aid in the application of the elementary principles assumed. So far as they will go in explanation of the *modus operandi* of cold in the production of particular diseases; so far as I shall be successful in my attempt, I shall, in fact, have furnished the inquirer with the data required for the solution of his etiological problem; so that little will be required from me under the next head, viz. *Diseases of cold.* Indeed, a detailed development of my views respecting the causation of those diseases would be doubly objectionable, namely, for the reason just given, and for the additional one, that the detail would necessarily involve much repetition of matter previously inserted under the present head of the *Morbific properties of cold.*

An *increased sensibility in the skin to friction, percussion,* &c. is an uniform effect of the cold bath: I have often observed it. This exaltation of sensibility is accompanied by augmented redness and vascularity, and is still more striking in the case of parts exposed to a biting atmosphere; and, though I have not myself witnessed it, I have no doubt but Macquart is within bounds when he says that "the slightest blow upon a hand or face frozen, as the popular expression is, with cold, excites a most violent sensation, and is often sufficient to cause rupture of the vessels, contusion, &c."

*Diminution of sensibility, with functional torpor,* from cold, are common effects of cold. When steadily applied, as in winter or in a protracted immersion in a cold bath, cold never fails to check the activity of the capillaries and exhalents of the skin, and transitory cold has the same effect in the predisposed. In the aphorisms of Sanctorius, (sect. 2, aph. 9,) we are informed that "if, in a warm season, a cold day happen, in the space of that day, supposing the way of living to be the same, about one-third part of the perspirable matter will be obstructed." The secreting and excretory processes are suspended; the oily and saline, and perhaps gaseous, ingredients of the perspiration, which are products of organic action, are, to a greater or less extent, wanting; the skin becomes dry and scaly; and the effete matters that should find their egress at the external surface are repelled towards the interior, where they must find some other outlet, or are detained in the circulating mass, to vitiate that source of all nutriment, and sow the seed of disease and death.

One of the first effects of cold on the skin, though last in this enumeration, is *contraction or shrinking,* which causes that appearance popularly called gooseskin: it is attended by an acumination of the bulbs of the hairs, and by an erection of the hairs themselves, which gives a disagreeable roughness and coarseness to the surface affected. In an intensely cold atmosphere, this constriction has been known to proceed to such an extreme as to produce cracking and laceration of the skin, as from excessive tension.

*Mucous surfaces.* The mucous surfaces, with the exception of that of the lungs and air-passages, are not exposed to the action of cold, and are seldom the seats of disease from that cause, except mediately and in consequence of some morbific impression transmitted to them from the skin. The following passage sufficiently proves that, in healthy subjects, the pulmonary mucous membrane is but little susceptible of derangement from cold.

"Exposure to a cold atmosphere, when the body is well clothed, produces no bad effect whatever, beyond a frost-bitten cheek, nose, or finger. As for any injury to healthy lungs from the breathing of cold air, or from sudden changes from cold into a warm air, or *vice versa*, it may, with much confidence, be asserted that, with due attention to external clothing, there is nothing in this respect to be apprehended." Captain Parry, whom I quote, (3d Voyage,) speaks from observation of the effects of extreme cold, and of violent and sudden transitions, on the health of 120 persons during four winters in polar regions, in which transition of from ninety to one hundred degrees in a space of less than half a minute were events of daily and hourly occurrence. He further says, that, under such circumstances, or previously to such transitions, "a covering for the mouth is not at all necessary, though very comfortable," (chap. 3.) Every one has experienced the safety, and even benefit, of iced drink, ices, &c. The mucous membranes, therefore, partly from being inaccessible (*ex. gr.* those of the abdomen and pelvis) to the air, partly, perhaps, from superior natural hardiness, (if such an expression is admissible,) are little liable to the diseases of cold from direct refrigeration, and suffer generally from atmospherical influence but mediately, and in consequence of disturbance of the cutaneous functions, suppression of the perspiration, and vicarious irritation.

The importance of that antagonism or disposition to vicarious action between the internal and external surfaces, which I have just alluded to as a source of disease in the mucous membranes from cold impressions on the skin, is very great. It has been before stated that the pulmonary tegument, with its ramifications in the face and pharynx, is the only mucous covering that can be immediately exposed to atmospherical influence; the only organ in fact, the skin excepted, that can suffer from cold directly, and without an exertion of the circuitous morbific power of some sympathetic connexion with the skin. It has also, in effect, been stated that, in the majority of cases, catarrhal affections of the air-passages are generally not owing to the inspiration of cold air, but to disturbed cutaneous action and suppressed perspiration. Now the mortality from cold is enormous, and arises not from cutaneous or mucous diseases solely, or even principally, but from inflammation, and other morbid affections of the serous coverings and substance of the viscera of the great cavities. The importance, then, of duly-sustained functional action in the skin, not only to the health of the mucous membranes, but to that of the frame in general, is very great. I am almost convinced with Ritter that he who takes adequate precautions against diseases of cold, avoids nine out of every ten disorders that he would have been liable to; and I consider his suggestion by no means absurd, when he half seriously, half jocosely, recommends that the following adage should be written over every door in Germany, in letters of gold, one foot long, "Behüte deine ausdünstung, auf das du lange lebest und gesund seyest."*

A curious morbid effect of the cold bath, and of a cold atmosphere, is frequent micturition, dependent apparently on irritation of the mucous membrane of the bladder. In some instances, the influence of cold applied to the external surface in producing irritation in the urinary organ strikingly illustrates what I have said relative to the influence of cutaneous refrigeration over the internal parts, and particularly the mucous tegument of the urinary passages. MARCARD mentions the case of a gentleman, who had borne with impunity the severity of a Russian winter, yet could not apply the palm of his hand to a cold stone without a desire to make water and an expulsive effort of the bladder.

Bulimia is another curious example of the morbid effect of cutaneous refrigeration: examples of it are numberless. PLUTARCH, in his life of Marcus Brutus, mentions that that remarkable person, having made a forced march, in order to reach Dyrrhachium (a town on the coast of Illyricum opposite Brundusium,) before Caius, Anthony's brother, had left his sutlers behind, owing to the ruggedness of the way and depth of the snows, and was seized with the disorder called Bulimia, or violent hunger, occasioned by cold and fatigue. Brutus growing very faint, and no provisions being at hand, his servants were forced to go to the gates of the enemy, to beg bread of the sentinels. He also says, that this disease affects both men and cattle after fatigue in the snow. BEAUPRÉ says that, in the retreat of the French army from Moscow, many fell victims to a frightful bulimia. In that unhappy retreat, the suppression of the cutaneous evacuations was followed by the most destructive derangements of the interior teguments. "The skin," says Beaupré, became dry, discoloured, filthy, earthy, and as if contracted. The suspension of the excretions of the skin was attended, as usual, by excessive and morbid activity of the mucous surfaces. From the month of November, the excessive cold had benumbed the vital energy of the skin, whilst that of the mucous membranes received a considerable increase, and such as characterizes inflammatory affection of that system; consequently, during the winter of 1812, Wilna, where great numbers of the French sick were deposited, and in the vicinity of which the

* "Guard well thy perspiration, that thou mayst live long and be healthy."

inhabitants, having lost their houses, had been equally exposed to cold and privation with the French army,—Wilna was infested with an epidemic catarrhal fever.

*Muscular system.* The morbid effects of cold on the muscles are *trembling, stiffness, cramps* or *spasms, instantaneous rigidity.*

The sensible part of the condition of *trembling* has its seat, no doubt, in the muscles: the quivering motion of the muscular fibre is, however, I suspect, but a part of the morbid effect of cold: I am disposed to consider it as itself an effect of an anterior morbific impression on the cutaneous nerves in which the motor nerves participate. To give my reasons for this opinion at length would occupy too much space. HUMBOLDT always found that cold applied to the substance of a muscle was followed, not by tremor, but by quiescence. "Versuche über die gereitzte Nerven-und Muskelfaser, &c." Like trials have, in some experiments of my own on the heart, been attended by like phenomena.

Stiffness and inaptitude to motion is a constant effect of protracted exposure in the cold bath: I have often experienced it. Of this state, the rigidity above mentioned amongst the effects of cold on the muscles, is but an exaggeration. QUINTIUS CURTIUS, in his "Life of Alexander," mentions, (speaking of the sufferings of the army in a wild northern expedition,) "In this uncultured wild, the destitute army has every variety of ill to endure: the blast of the desert extinguished life in many, and caused the feet in others to mortify; its white glare perniciously affected the eyes of the majority; some, having stretched on a bed of ice their exhausted frames, were, through want of motion, so stiffened by the frost that, when they tried to rise, they were unable; the torpid were lifted up by their comrades. There was no better remedy than compelling to walk," he continues; "the vital heat thus excited, the use of the limbs in part returned."

"In the Russian campaign," says Beaupré, "soldiers have been seen expiring instantaneously, as if thunderstruck from excessive cold. At Smolensko the temperature was so rigorous, that more than thirty grenadiers of the Italian guard fell frozen as they attempted to set themselves in line on a height beyond the Borysthenes. A battalion of the regiment I belonged to, when encamped on the same height, lost in this way many men in a single day." In such cases, as also in examples of persons suddenly immersed in very cold water after violent exercise, as skaiters, instantaneous death is principally attributable to asphyxia produced by fixation of the chest, owing to spasmodic rigidity and rapid congelation of the muscles of the thorax. In some cases, also, apoplexy or syncope might be supposed.

The Cramp that attacks swimmers is an effect of cold on the muscular system, with which every one is familiar. Dr. CURRIE informs us, that "the principal source of inconvenience and pain experienced by Mr. Amyot, in his perilous situation after the shipwreck in the mouth of the Mersey, in December 1790," (of which Dr. Currie has published an account,) "was spasms of the voluntary muscles: what most distressed him were cramps in the muscles of his sides and hips, which were drawn into knots." We are informed by MM. PARAT and MARTIN, (membres de la Société Méd. de Lyons,) that the French soldiers that inhaled incautiously the piercing blasts of Mont St. Bernard, during Napoleon's march across the mountains into Piedmont, were in numerous instances affected with spasmodic contraction of lips, contractions of the fanus, and other similar affections of the air-passages. Captain SCORESBY mentions, in his "Arctic Regions," that on one morning, after a night during which the thermometer had fallen from thirty to fifteen degrees (Fahr.), the following effects were observed: the circulation of the blood was accelerated; a sense of parched dryness was excited in the nose: the mouth, or rather lips, were contracted as if by a sphincter, and the articulation of many words rendered difficult and imperfect.

*Blood.* The blood is, perhaps, after all, that constituent of the animal that suffers, in many cases, the most important modifications from cold. The blood is a current into which pass all the effete particles, on their way to the several emunctories; it is likewise the source of all materials deposited by nutrient arteries in the substance of the organs. Every accession and every loss to its tide is preceded or followed by chemical change, and to every chemical change the presence of caloric, in some certain quantity, must be essential. To the continuance, therefore, of the higher functions of the blood, the application of cold, i. e. the abstraction of heat, must, to a greater or lesser extent, prove an obstacle. The effects, therefore, of refrigeration on the blood, considered merely in its chemical relations to the vital organs, must be of great importance. The share, therefore, of the blood in the constitution of the majority of diseases of cold, (*ex. gr.* in inflammatory and cachectic diseases,) must, however indefinite and unascertained, be very considerable. And the extent of the morbific power would be greatly enlarged if, in addition to the chemical affections just alluded to, we should, with G. R. TREVIRANUS, Dr. EDWARDS, and others, attribute to the red globules a sort of independent vitality. Though not a convert to that physiological theory, on whichever side truth may lie, I consider the facts and reasonings of Dr. Treviranus ("Biologie") extremely interesting, and readily acknowledge the importance of the observations of Dr. Edwards, to the accuracy of part of which, through the favor of that gentleman, I can myself bear witness.

The morbid changes in the vital fluid above alluded to are Plethora, the Inflammatory state, and the Degenerations of cachexies, fevers, &c.

Plethora from cold seasons and weather arises partly from diminished waste, and partly from increased supply: there is in healthy persons, in winter, a great diminution in the evacuation by the skin, which is by no means compensated for by increased activity of the other emunctories. Sanctorius found that temperate persons weigh about three pounds more in winter than in summer. The indolent and luxurious experience, in many cases, great augmentation, both in size and weight, towards the end of the cold season. In winter, the expenditure, in the form principally of perspiration, not only is greatly less, but the appetite, powers of digestion, and consequently supply of nutrient matter, are often considerably greater than in summer.

The *inflammatory state of the blood,* marked by the formation of the "buffy coat" on blood drawn by the lancet, is the effect, probably, of some reaction of the nervous system on the vascular, and on its contents: that state may, I believe, exist without local inflammation. It is not a normal state in any population, however far from the line; nor does cold weather produce it simply as cold weather. It has been supposed that the state of the blood under consideration is produced by the matter of the suppressed perspiration, which, being retained in the circulation, vitiates the fluids, and communicates to them that morbid disposition. It has been further proposed as a solution of the question why some persons more readily catch cold than others, that the former class excrete a more irritating, and as it were poisonous, perspiration; while the comparative immunity enjoyed by the latter is owing to the blander and more innocuous composition of their perspiration. But I have little faith in either of those speculations. Another opinion that I have met with is still less probable, viz. that the cold coagulates, as it were, the albuminous matter, and condenses it (as I understand the writer) so as to render it concrescible on the abstraction of heat and motion. The ætiology, in truth, of "buffy blood" is quite uncertain. An infant physiology leaves us almost entirely in the dark respecting the normal susceptibilities of the blood. We have nothing like adequate information regarding the relation between that fluid and the nervous system more particularly, which is the very quarter probably in which further light would be most useful. There is, on the one hand, good reason to suppose that the blood is capable of assuming the inflammatory state, independently of any morbific operation on the part of the nervous organs: plants, insects, brainless monsters, paralytic limbs, &c. are susceptible of inflammation. Nor are facts of an opposite tendency wanting. Irritation of nerve by mechanical stimulus is capable of producing turgescence and inflammation, with all its consequences, in a part previously healthy. The nervous system is in the highest degree sensible to change of temperature: there is no direct or unequivocal proof that the blood is, like the nerves, susceptible of morbid changes from atmospherical influence. The source of that animal heat, also, which figures so prominently in diseases of cold, may be the blood alone or the nerves alone; or it may be the product of conjoint action, the blood furnishing the material, and the nerves the stimulant. With such an imperfect knowledge of the relations between the blood and physical agents, and between the blood and the nervous system, it is impossible that the ætiology of diseases of cold should not to a great extent be a mystery.

Another morbid state, or class of morbid states, above referred to, are those *cachectic* conditions of the blood that characterize many of the chronic diseases of cold, *ex.gr.* scurvy, jaundice, chlorosis, and other obscure affections in many cases attributable to cold. Exposure to cold has frequently occasioned icterus and suppressio mensium. Amenorrhœa and scorbutus are diseases most frequent in cold latitudes. In those and other cachectic states, febrile or otherwise, there can be no doubt of the presence of an unhealthy crasis of the blood: the following picture, though a sketch of an extreme case, will illustrate the operation of cold seasons in vitiating the blood, by preventing depuration at the emunctories, and favoring stagnation, degeneration, and perhaps decomposition. "The want of a constant supply of warmth is here (in polar regions) immediately followed by a condensation of all the moisture, whether from the breath, victuals, or other sources, into abundant drops of water, very rapidly forming on all colder parts of the deck. A still lower temperature modifies, and perhaps improves, the annoyance, by converting it into ice; which again an occasional increase of warmth dissolves, by converting it into water. Not only is a moist atmosphere thus continually kept up, but it is rendered foul by the want of that ventilation which warmth alone can furnish. With an apartment in this state, the men's clothes and bedding are continually in a moist and unwholesome condition, generating a deleterious air, which there is no circulation to carry off; and whenever these circumstances combine for any length of time together, so surely may the scurvy, to say nothing of other diseases, be confidently expected." (*Parry, 3d Voyage.*)

(To be continued.) Vol. lxviii. p. 21.

---

